# Dipeptidyl Peptidase-4 Inhibitors Use and Relative Risk of Ischemic Cerebrovascular Disease in Type 2 Diabetic Patients in a Case-Control Study

**DOI:** 10.3389/fphar.2017.00859

**Published:** 2017-11-22

**Authors:** Shih-Wei Lai, Kuan-Fu Liao, Cheng-Li Lin, Hsien-Feng Lin

**Affiliations:** ^1^Department of Medicine, China Medical University, Taichung, Taiwan; ^2^Department of Family Medicine, China Medical University Hospital, Taichung, Taiwan; ^3^Department of Medicine, Tzu Chi University, Hualien, Taiwan; ^4^Department of Internal Medicine, Taichung Tzu Chi General Hospital, Taichung, Taiwan; ^5^Management Office for Health Data, China Medical University Hospital, Taichung, Taiwan; ^6^Department of Chinese Medicine, China Medical University, Taichung, Taiwan

**Keywords:** diabetes mellitus, DPP-4 inhibitors, ischemic cerebrovascular disease, National Health Insurance Program, Taiwan

## Abstract

**Background and Objectives:** Limited research focuses on the risk of ischemic cerebrovascular disease associated with use of dipeptidyl peptidase-4 inhibitors (DPP-4 inhibitors) in patients with type 2 diabetes mellitus in Taiwan. This study aimed to investigate the association between DPP-4 inhibitors use and the first episode of ischemic cerebrovascular disease.

**Methods:** We designed a case-control study using the database of the Taiwan National Health Insurance Program. There were 1999 type 2 diabetic subjects aged 20–84 years with the first episode of ischemic cerebrovascular disease from 2000 to 2013 as the cases, and 7996 sex- and age-matched, randomly selected type 2 diabetic subjects aged 20–84 years without any type of cerebrovascular diseases as the matched controls. We estimated the odds ratio (OR) and 95% confidence interval (CI) of ischemic cerebrovascular disease associated with cumulative duration of DPP-4 inhibitors use by the multivariable logistic regression model.

**Results:** After adjustment for confounding variables, the adjusted OR of ischemic cerebrovascular disease was 0.96 (95% CI 0.95, 0.97) in subjects with ever use of DPP-4 inhibitors as increase in use duration for every 1 month, compared with never use. The sub-analysis disclosed that the adjusted ORs of ischemic cerebrovascular disease were 1.57 (95% CI 1.36, 1.80) for subjects with cumulative duration of DPP-4 inhibitors use <1 year, and 0.70 (95% CI 0.57, 0.87) for subjects with cumulative duration of DPP-4 inhibitors use ≥1 year, compared with never use.

**Conclusion:** Our findings suggest that DPP-4 inhibitors use correlates with relative risk reduction of the first episode of ischemic cerebrovascular disease in type 2 diabetic patients in a duration-dependent response. The beneficial effect will be marked when DPP-4 inhibitors use is ≥1 year.

## Introduction

Dipeptidyl peptidase-4 inhibitors (DPP-4 inhibitors) are classified as a new oral anti-diabetic medication and are widely used for management of type 2 diabetes mellitus (Baetta and Corsini, 2011). In addition to their glucose-lowering effects, current evidence discloses that DPP-4 inhibitors have beneficial effects on the major adverse cardiovascular events (Monami et al., 2011; Scheen, 2013; Davidson, 2014; Kaneko and Narukawa, 2016).

Cerebrovascular disease ranked the fourth leading cause of death and diabetes mellitus ranked the fifth leading cause of death in Taiwan in 2016, respectively (Ministry of Health and Welfare, 2017a). To date, conclusive results on the risk of ischemic cerebrovascular disease associated with DPP-4 inhibitors use in Taiwan are limited. If the association between the risk of ischemic cerebrovascular disease and DPP-4 inhibitors use substantially exists, the drug of choice for management of type 2 diabetes mellitus can be suggested in Taiwan. Therefore, we designed a case-control study using the database of the Taiwan National Health Insurance Program to investigate the association between DPP-4 inhibitors use and the first episode of ischemic cerebrovascular disease.

## Materials and Methods

### Study Design and Study Population

The methodology was adapted from previous studies (Lai et al., 2016, 2017j; Liao et al., 2017b). It did not need to write published protocol details. We summarized them as follows and cited the relevant references. We designed a case-control study using the database of the Taiwan National Health Insurance Program. Taiwan is an independent country with more than 23 million persons (Chan et al., 2016; Chang and Yu, 2016; Chang et al., 2016; Chen and Wu, 2016; Chen S.Y. et al., 2016; Chen Y.F. et al., 2016; Hsieh et al., 2016; Hsu and Yin, 2016; Huang and Chang, 2016; Lin and Lin, 2016; Maa and Leu, 2016; Ooi, 2016; Yu et al., 2016; Lai et al., 2017g; Lee et al., 2017; Liang et al., 2017; Liao et al., 2017a,c; Lin et al., 2017a; Liu et al., 2017; Wen and Yin, 2017; Wu et al., 2017; Yang J.S. et al., 2017; Yang M.D. et al., 2017). The program launched in March 1995, and has covered nearly 99.6% of 23 million persons at the end of 2015 (Ministry of Health and Welfare, 2017b). The program details have been described in previous studies (Lai et al., 2010; Chen H.Y. et al., 2016; Tsai et al., 2016; Chu et al., 2017). The Research Ethics Committee of China Medical University and Hospital in Taiwan approved the study (CMUH-104-REC2-115).

### Inclusion Criteria

We selected type 2 diabetic subjects aged 20–84 years with the first episode of ischemic cerebrovascular disease as the cases during the period of 2000–2013 based on International Classification of Diseases, 9th Revision, Clinical Modification (ICD-9 codes 433, 434, and 435). The date of a subject being diagnosed with the first episode of ischemic cerebrovascular disease was defined as the index date. Additionally, for every one case with ischemic cerebrovascular disease, approximately four type 2 diabetic subjects aged 20–84 years without any type of cerebrovascular diseases were randomly selected as the matched controls. The cases and the matched controls were matched with sex, age (5-year interval), comorbidities, and the year of index date.

### Selection of Comorbidities

Comorbidities potentially related to ischemic cerebrovascular disease before index date were identified as follows: alcohol-related disease, atrial fibrillation, chronic kidney disease, chronic obstructive pulmonary disease, coronary artery disease, hyperlipidemia, and hypertension. Based on the ICD-9 codes, the diagnosis validity of comorbidities was discussed in previous studies (Lai et al., 2017a,b,c,d,e,f; Liao et al., 2017e; Lin et al., 2017b,c).

### Definition of DPP-4 Inhibitors Use and Other Anti-diabetic Medications Use

Prescription histories of medications studied were collected. Other anti-diabetic medications on Taiwan market during 2000–2013 were metformin, sulfonylureas, α-glucosidase inhibitors, thiazolidinediones, and insulins. The definition of medications use was adapted from previous studies (Cheng et al., 2017; Lai et al., 2017h,i; Liao et al., 2017d,f). Briefly, subjects with at least a prescription for medications studied before index date were classified into “ever use.” Subjects without a prescription of medications studied before index date were classified into “never use.”

### Statistical Analysis

At first, we made a comparison of sex, age, medications, and comorbidities between the cases and the matched controls by using the Chi-square test for categorized variables and the *t*-test for continuous variables. Then, variables which were significantly associated with ischemic cerebrovascular disease in the univariable logistic regression model were further tested by the multivariable logistic regression model. We estimated the odds ratio (OR) and 95% confidence interval (CI) for the relative risk of ischemic cerebrovascular disease associated with cumulative duration of DPP-4 inhibitors use. The probability value < 0.05 was considered statistically significant (SAS software version 9.2, SAS Institute, Inc., Cary, NC, United States).

## Results

### Basic Characteristics of the Study Population

**Table 1** discloses the basic characteristics of the study population. There were 1999 cases with the first episode of ischemic cerebrovascular disease in 2000–2013 and 7996 matched controls without any type of cerebrovascular diseases, with similar distributions of sex and age. The mean ages (standard deviation) were 67.8 (10.6) years in cases and 67.7 (10.6) years in matched controls, without statistical significance (*t*-test, *P* = 0.67). The cases were more likely to have higher proportions of ever use of DPP-4 inhibitors, ever use of other anti-diabetic medications, alcohol-related disease, atrial fibrillation, chronic kidney disease, coronary artery disease, hyperlipidemia, and hypertension than the matched controls, with statistical significance (Chi-square test, *P* < 0.05, for all).

**Table 1:** Basic characteristics between cases with ischemic cerebrovascular disease and matched controls.

	Matched controls *N* = 7996		Cases with ischemic cerebrovascular disease *N* = 1999	
Variable	*N*	(%)	*N*	(%)	*P*-value^∗^
Sex					0.99
Female	3580	(44.8)	895	(44.8)	
Male	4416	(55.2)	1104	(55.2)	
Age group (years)					0.99
20–49	496	(6.2)	124	(6.2)	
50–64	2500	(31.3)	625	(31.3)	
65–84	5000	(62.5)	1250	(62.5)	
			
Age (years), mean ± standard deviation^†^	67.7 ± 10.6		67.8 ± 10.6		0.67
Ever use of DPP-4 inhibitors	1485	(18.6)	446	(22.3)	<0.001
Ever use of other anti-diabetic medications	7741	(96.8)	1960	(98.1)	0.003
Comorbidities before index date					
Alcohol-related disease	720	(9.00)	209	(10.5)	0.046
Atrial fibrillation	220	(2.75)	158	(7.90)	<0.001
Chronic kidney disease	1602	(20.0)	441	(22.1)	0.04
Chronic obstructive pulmonary disease	2617	(32.7)	597	(29.9)	0.01
Coronary artery disease	3586	(44.9)	948	(47.4)	0.04
Hyperlipidemia	5697	(71.3)	1480	(74.0)	0.04
Hypertension	6517	(81.5)	1830	(91.6)	<0.001


*Data are presented as the number of subjects in each group with percentages given in parentheses. ^∗^Chi-square test and ^†^*t*-test comparing subjects with and without ischemic cerebrovascular disease*.

### Relative Risk of Ischemic Cerebrovascular Disease Associated with Cumulative Duration of DPP-4 Inhibitors Use

After adjustment for confounding variables including other anti-diabetic medications, alcohol-related disease, atrial fibrillation, chronic kidney disease, chronic obstructive pulmonary disease, coronary artery disease, hyperlipidemia, and hypertension, the adjusted OR of ischemic cerebrovascular disease was 0.96 (95% CI 0.95, 0.97) in subjects with ever use of DPP-4 inhibitors as increase in use duration for every 1 month, compared with never use. The sub-analysis disclosed that the adjusted ORs of ischemic cerebrovascular disease were 1.57 (95% CI 1.36, 1.80) for subjects with cumulative duration of DPP-4 inhibitors use <1 year, and 0.70 (95% CI 0.57, 0.87) for subjects with cumulative duration of DPP-4 inhibitors use ≥1 year, compared with never use (**Table 2**).

**Table 2:** Relative risk of ischemic cerebrovascular disease associated with cumulative duration of DPP-4 inhibitors use.

Variable	Case number/control number	Crude OR	(95% CI)	Adjusted OR^†^	(95% CI)
Never use of DPP-4 inhibitors as a reference	1553/6511	1.00	(Reference)	1.00	(Reference)
Cumulative duration of DPP-4 inhibitors use (increase in use duration for every 1 month)	446/1485	0.96	(0.95, 0.98)	0.96	(0.95, 0.97)
Cumulative duration of DPP-4 inhibitors use					
<1 year	340/880	1.62	(1.41, 1.86)	1.57	(1.36, 1.80)
≥1 year	106/605	0.74	(0.59, 0.91)	0.70	(0.57, 0.87)


^†^*Variables found to be statistically significant in the univariable logistic regression model were further tested by the multivariable logistic regression model. Only other anti-diabetic medications use, alcohol-related disease, atrial fibrillation, chronic kidney disease, chronic obstructive pulmonary disease, coronary artery disease, hyperlipidemia, and hypertension could be further tested*.

## Discussion

In this case-control study, we found that DPP-4 inhibitors use was associated with decreased odds of the first episode of ischemic cerebrovascular disease in type 2 diabetic patients in a duration-dependent response. The effect would be marked when DPP-4 inhibitors use was ≥1 year. This finding was consistent with previous studies disclosing that DPP-4 inhibitors use was associated with lower risk of ischemic cerebrovascular disease (Ou et al., 2015; Shih et al., 2016). We found that if DPP-4 inhibitors use was less than 1 year, the odds would be high (adjusted OR1.57). That is, short-term of DPP-4 inhibitors use does not have the beneficial effect. Clinicians should keep in mind about the risk of ischemic cerebrovascular disease during the first year of DPP-4 inhibitors use in type 2 diabetic patients. We suggest that only long-term of DPP-4 inhibitors use for 1 year or longer, type 2 diabetic patients can have the beneficial effect on risk reduction of ischemic cerebrovascular disease.

Hemoglobin A1c is an important indicator for long-term glycemic control. Previous studies disclosed that high levels of hemoglobin A1c were associated with increased risk of cerebrovascular disease (Goto et al., 2015; Cavender et al., 2016). Although DPP-4 inhibitors are not the drug of first choice for type 2 diabetes mellitus by the guidelines of American Diabetes Association (American Diabetes Association, 2017). DPP-4 inhibitors use still can reduce hemoglobin A1c by 0.5–1% (Madsbad et al., 2008; Grunberger, 2014). In our opinion, patients using other anti-diabetic medications but poor glycemic control would additionally use DPP-4 inhibitors. When initiating DPP-4 inhibitors, Hemoglobin A1c levels of these patients should be high. The risk for ischemic cerebrovascular disease was still greater. Only using DPP-4 inhibitors for 1 year or longer and then hemoglobin A1c gradually reducing, these patients could have a chance to be in good glycemic control. Thus, the risk of ischemic cerebrovascular disease was further reduced. That at least partially explains why the odds of ischemic cerebrovascular disease would be high during the first year of DPP-4 inhibitors use, and then the odds would be reduced after 1 year.

Some limitations should be mentioned. First, due to the inherent limitation, hemoglobin A1c was not recorded in the database. Our study could not prove the association between the risk of ischemic cerebrovascular disease and hemoglobin A1c levels. Second, **Table 1** discloses that about 97% of study subjects had ever used other anti-diabetic medications. It is difficult to investigate the absolute risk of ischemic cerebrovascular disease associated with DPP-4 inhibitors use alone due to the small eligible number for DPP-4 inhibitors use alone. The rational option was to investigate the relative risk of ischemic cerebrovascular disease associated with DPP-4 inhibitors use after adjustment for other anti-diabetic medications. Third, a case-control study could not prove the causal relationship. Further prospective cohort research is warranted to focus on the association between the absolute risk of ischemic cerebrovascular disease and DPP-4 inhibitors use alone.

Some strengths should be emphasized. The title clearly and precisely reflects the findings of the study. The statistical methods are used validate. The prior works are properly and fully cited. Immortal time bias can be minimized in a case-control study. The results are convincing.

## Conclusion

Our findings suggest that DPP-4 inhibitors use correlates with relative risk reduction of the first episode of ischemic cerebrovascular disease in type 2 diabetic patients in a duration-dependent response. The beneficial effect will be marked when DPP-4 inhibitors use is ≥1 year.

## Ethics Statement

Insurance reimbursement claims data used in this study were available for public access. Patient identification numbers had been scrambled to ensure confidentiality. Patient informed consent was not required.

## Author Contributions

S-WL and K-FL contributed to the conception of the article, initiated the draft of the article, revised the article, and contributed equally to the article. C-LL conducted the data analysis and reviewed the article. H-FL participated in the data interpretation and revised the article.

## Conflict of Interest Statement

The authors declare that the research was conducted in the absence of any commercial or financial relationships that could be construed as a potential conflict of interest.
